# OTEH-11. Single Cell RNA Sequencing to identify cellular heterogeneity with in Pituitary Adenomas

**DOI:** 10.1093/noajnl/vdab070.050

**Published:** 2021-07-05

**Authors:** Saket Jain, Husam Babikir, karin Shamardani, Aaron Diaz, Manish Aghi

**Affiliations:** University of California San Francisco, San Francisco, CA, USA

## Abstract

Pituitary adenomas (PA) are one of the most common primary brain tumors and comprise approximately 15% of brain neoplasms. Most PA are histologically benign, but can cause significant morbidity. Previous studies utilized whole genome and exome sequencing to identify a few somatic variants, but no recurrent mutations were observed. Further studies are warranted to identify driver mutations occurring at low frequencies. We used single-cell RNA sequencing (10X Genomics) to investigate cellular heterogeneity in 12 non-functioning pituitary adenomas. Our analysis identified discrete clusters of cells associated with specific functional pathways. One of these clusters corresponded to cells expressing genes related to metabolic pathways, primarily lipid metabolism. Another cluster consistent amongst the three patients comprised cells involved in antigen presentation and processing. In addition, the copy number variation analysis highlighted distinct chromosomal alterations within our samples. Interestingly, we were able to identify clonal variations within each tumor based on chromosomal aberrations. For example, in our first patient we observed a gain of chromosome 19 and loss of chromosome 2. Our analysis showed three different clonal populations within this tumor. All three populations harbored the loss of chromosome 2, one population exhibited gain of chromosome 19, while a third population exhibited loss of chromosome 19. These early results indicate the loss of chromosome 2 as an early event in tumorigenesis and gain/loss of chromosome 19 as late events. We are currently in a process of identifying somatic variations within these tumors by variant calling. Currently we are expanding our analysis to 20 non-functional PA. Mapping the single-cell gene expression profiles with mutational phylogeny will reveal the differences in clonal evolution within the tumor subtypes. This study will help us define the molecular fingerprint of pituitary adenomas and provide insights which could be utilized in the clinic for better management of these tumors.

